# Optical Properties of Crystalline Lactose Fluidized with Dilutions of Various Substances in the Terahertz Frequency Range

**DOI:** 10.3390/pharmaceutics14010032

**Published:** 2021-12-24

**Authors:** Anna Baranova, Anastasiya Lykina, Daria Antonova, Olga Smolyanskaya

**Affiliations:** Femtomedicine Laboratory, ITMO University, 197101 Saint-Petersburg, Russia; anna.baranova@itmo.ru (A.B.); dantoshka@list.ru (D.A.); smolyanskaya@itmo.ru (O.S.)

**Keywords:** lactose, tablets, terahertz spectroscopy, principle component analysis, control of crystal structure, new properties

## Abstract

Lactose is a commonly used component of pharmaceutical medications in tablet form. It was previously shown that lactose changes conformationally after saturation in fluidized beds with active pharmaceutical ingredients obtained by repeated dilution of antibodies to interferon-gamma in combination with an external intensive vibration treatment. Moreover, it was revealed that these solutions are self-organized dispersed systems in which nano-objects are formed. Their biological activity and mechanism of action were previously established as well. The current work was dedicated to investigating the optical properties of fluidized lactose powders in the terahertz frequency range. Spectral analyses of powders of crystalline lactose saturated in fluidized beds with a diluted solution of either glycine buffer, antibodies to interferon-gamma, or water were carried out, intact lactose served as a control. All powders were tableted before testing. In the course of the study, the macroscopic parameters of the tablets were established, at which they had a stable shape and their THz optical properties had no parasitic diffraction losses. These tablets were analyzed using terahertz time-domain spectroscopy in the frequency range of 0.2–2.6 THz. The differentiation between the spectra was conducted using a principal component analysis. The differences between intact lactose and the lactose saturated with any of studied solutions were demonstrated. Additionally, lactose saturated with solutions of multiple dilutions of a substance (antibodies or glycine buffer) differed not only from intact lactose, but also from lactose saturated with a diluted solution of water. Moreover, discrimination of lactose formulations saturated with different substances (antibodies or glycine buffer) was also possible. Additionally, intact lactose differed from lactose saturated with diluted water. The methods reported could be useful for the quality control of the medications based on the technology of repeated dilution of an original substance.

## 1. Introduction

Lactose is a milk disaccharide carbohydrate that is also found as so-called “hidden lactose” in nondairy products (e.g., processed meat) and has to be controlled and labeled due to high worldwide rates of lactase deficiency [1]. Also, lactose is a commonly used component of pharmaceutical medications in tablet form. The structure of lactose triturated with an active pharmaceutical ingredient differs from the pure crystalline lactose; it is due to the destruction and creation of new chemical groups in lactose and modification of its crystal structure [2]. In [3], lactose conformation was changed by saturation in fluidized beds with active pharmaceutical ingredients obtained by repeated dilution of antibodies (Abs) to interferon-gamma (IFN-γ) in combination with an external intensive vibration treatment between the dilution steps. Moreover, it was revealed that these solutions are self-organized dispersed systems in which nano-objects are formed [4]. It was also observed that the ingredient trituration process in lactose powder is accompanied by the formation of micro- and nano-particulates [2]. Their biological activity and mechanism of action were previously established [5], and insights into the molecular mechanism of their action were gained [6].

Lactose saturated with dilutions of substances can be distinguished from the control lactose sample using UV spectroscopic methods [7,8,9,10]. However, spectroscopy in the far-infrared frequency range is of interest as it allows to obtain information on the structural properties of various biochemical compounds, since many vibrational and rotational modes of molecules are in the 0.1–10.0 terahertz (THz) range. Therefore, it is crucial to study the spectral characteristics of lactose monohydrate containing various concentrations of substances in the THz range. Such studies would allow to understand how presence of the substances affects the structure of lactose.

Terahertz time-domain spectroscopy (THz-TDS) is a preferable method for studying low-frequency modes of structures due to the high signal-to-noise ratio (SNR) and the ability to obtain the refractive index and absorption coefficient of the objects being studied [11].

The properties and spectral features of crystalline α-lactose monohydrate were previously studied. Several works [12,13,14] confirmed the presence of absorption spectral peaks in the 0.1 to 2.6 THz range at frequencies of 0.53 THz (a peak presumably corresponding to the hindered rotational mode of the α-lactose molecule in the crystal), 1.38, 1.82, and 2.56 THz. By using the THz spectroscopic analysis, it is also possible to observe structural changes in a substance in the absorption spectrum at different temperatures [15]. So far, studies on the spectral analysis of α-lactose monohydrate fluidized with dilutions of pharmaceutical ingredients in the far-IR range were not performed.

THz spectral data can be visually analyzed, and identification of samples by their specific spectral characteristics (i.e., peaks or absorption bands) is possible. Nevertheless, for additional control of THz spectral characteristics, some chemometric methods are actively used. Principal component analysis (PCA) is one of the most common chemometric analysis methods [16,17,18]. This analysis reveals correlations between different spectral characteristics; it can also be used to compress data. Thus, Watanabe et al. [16] and Shen [17] used the PCA method to determine the spatial distribution of various chemical compounds in tablet form, and Haware et al. [19] also applied the PCA method to quantify relationships between different powder samples and the compression properties of the tablets. The use of the PCA method for identifying the objects by their spectral properties can be effective even if they do not have characteristic features.

Lactose is an important component of drugs which active pharmaceutical ingredients are obtained by repeated dilution of antibodies. These drugs are effective and safe in the treatment of many diseases [20,21,22,23,24,25,26,27]. Although previous studies already showed the differences between lactose saturated with dilutions of substances from control [3,6,7,8,9,10], the development of comprehensive methods of quality control of such drugs is still an urgent task. Considering the advantages of THz spectroscopy, the goal of this work was, using this method and a PCA, to investigate the possibility of distinguishing lactose saturated with a substance in a low concentration from controls that do not contain the substance. Four formulations of lactose monohydrate powders were used as the studied objects: intact sample and crystalline lactose saturated with a diluted solution of glycine buffer (GB), a diluted solution of Abs to IFN-γ, or diluted water.

When studying the optical properties of pharmaceutical materials in the THz frequency range, the samples under study must meet certain requirements [28]. To avoid any parasitic diffraction losses in the THz optical properties, the tablets should be homogeneous and have a minimum and well-defined thickness and a certain density. Based on these requirements, at the first stage of the study, the macroscopic parameters of the tablets were selected, at which the tablets had a stable shape.

## 2. Materials and Methods

### 2.1. Lactose Formulations Preparation

The following formulations of lactose powder produced from lactose monohydrate (SuperTab® 30GR, DFE pharma, Goch, Germany) of the same batch were tested in the present study:

1. Lactose not subjected to any treatment is hereinafter referred to as “intact lactose”.
2. Lactose (lactose monohydrate) saturated with a solution of affinity-purified rabbit polyclonal Abs to IFN-γ that had previously undergone a process of gradual reduction of their initial concentration under specific conditions [29]. Briefly, Abs to IFN-γ (2.5 mg·mL${}^{-1}$) were mixed with aqueous ethanol solution at a 1:100 ratio with intensive vibration treatment to produce the first centesimal dilution. All subsequent dilutions contained 1 part of the previous dilution and 99 parts of the solvent. The final solution contained a mixture of 12, 30, and 50 centesimal dilutions of Abs to IFN-γ. The estimated theoretical Abs concentration in the final solution was no more than 2.5 × 10${}^{-24}$ mg·mL${}^{-1}$, or 2.5 × 10${}^{-18}$ ppb (w/w). However, according to the physicochemical studies, for the samples manufactured using the technology of high dilutions, such estimation might not be correct due to nonlinear reduction in solute concentration. Indeed, it was shown that even in dilutions lower than 10${}^{-24}$, the solute molecules can be preserved due to the flotation effect [30,31,32,33,34]. The original substance of the Abs was produced following the current EU requirements of Good Manufacturing Practice for starting materials [35] by AB Biotechnology (Edinburgh, UK). Hereinafter, lactose saturated with dilutions of Abs to IFN-γ is referred to as “Abs saturated lactose”.
3. Lactose treated using the same technology as above, but glycine buffer was taken as the initial solution for dilutions (instead of Abs to IFN-γ). This formulation is hereinafter referred to as “GB saturated lactose”.
4. Lactose treated using the same technology as the previous two, but deionized water, obtained using a Milli-Q water purification system (Merck Millipore, Darmstadt, Germany) with conductivity of 18.3 MΩ cm, was taken as the initial solution (instead of Abs to IFN-γ or glycine buffer). Namely, deionized water had undergone a process of gradual reduction of its initial concentration via mixing with aqueous ethanol solution, according to the procedure described above. This formulation is hereinafter referred to as “technological control”.

All the lactose powders tested were provided by OOO “NPF “MATERIA MEDICA HOLDING” (Moscow, Russia) in an encoded form (the formulations were decoded after all of the results were analyzed). The sampling of powders for the study was carried out following the internal procedure that meets the pharmacopoeial requirements for sampling of crude medications and medicinal products, ensuring their uniformity and excluding contamination. For THz optical properties determination of lactose monohydrate powders using THz-TDS in transmission mode, the formulations were weighed (accuracy about 0.01 mg, Analytical balance OHAUS Discovery, NJ, USA), placed in a steel mold, and pressed under a pressure of 245 MPa (Manual press Corvette-590, Enkor, Voronezh, Russia) for obtaining tablets with a diameter of 5 mm. The thickness of the tablets was measured using a micrometer (accuracy about 10 μm, digital micrometer Inforce 06-11-44, Inforce, Yaroslavl, Russia). The density of the tablets is calculated using the following equation:

(1) $$\rho_{0}=m\cdot {\left(\frac{\pi \cdot D^{2}}{4}\cdot d\right)}^{-1},$$

where *m* [mg], *d* [mm], and *D* [mm] stand for the mass, the thickness, and the diameter of a tablet respectively.

Thirty-six tablets were made to analyze the optical characteristics of lactose formulations using THz-TDS. Each 9 of these tablets contained intact lactose, GB saturated lactose, Abs saturated lactose, or technological control. The tablet thickness ranged from 0.91 to 3.33 mm, weight from 24.0 to 90.8 mg, and calculated density from 1.31 to 1.39 mg·mm${}^{-3}$.

### 2.2. Experimental Setup

Terahertz transmission spectra of the tablets were measured using a TeraPulse 3000 time-domain spectroscopy system from TeraView (UK). The system uses a Ti:Sapphire laser with a wavelength of 800 nm, a frequency of 80 MHz, and an optical pulse duration of 90 fs. The pump pulse is incident on a photoconductive emitter on a GaAs substrate to create electron-hole pairs, which are then accelerated by an electric field applied to the electrodes. During the relaxation of charge carriers, terahertz pulses of bandwidth of 0.1–3.0 THz are emitted. The THz radiation pulses pass through the sample and hit the photoconductive detector (similar to the emitter), which also is irradiated by the incident pulse of 800 nm 90 fs, obtained by splitting the beam of the previously mentioned laser after passing through the delay line. In this study, a transmission module installed in a special chamber of the THz spectrometer was used. The space through which the THz beam passes is purged with nitrogen to reduce the absorption caused by water vapor in the air.

The tablets were placed on a special holder in the vertical position. THz radiation was focused on one point on the tablets; five THz signals were recorded. To improve accuracy, the results were averaged and the standard deviation was calculated. This was done to reduce the effect of the noise component of the spectra when revealing the performances of the spectra of lactose monohydrate tablets.

**Figure 1:**
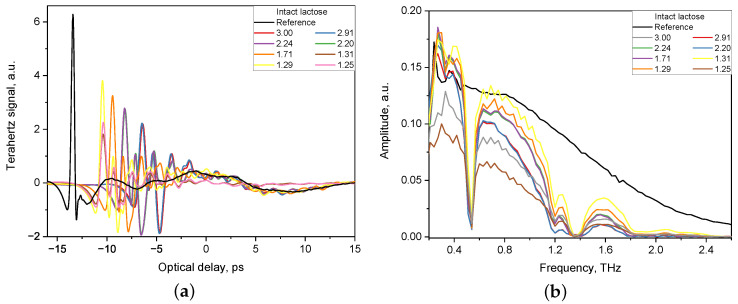
Reference signal and signal of tablets with various thickness (1.25–3.00 mm) obtained by THz-TDS system in time domain (**a**) and in frequency domain (**b**).

The acquired waveforms of the THz electric field for both the tablet and the reference were then converted to the frequency-domain by fast Fourier transformation. The THz-TDS system has built-in software that allows to extract the refractive index and the absorption coefficient of a particular specimen. Figure 1 shows an example of the THz pulses of THz-TDS system transmitted through tablets with intact lactose of various thickness (1.25–3.00 mm) and a reference signal.

### 2.3. Principal Component Analysis for Sample Identification

Analysis of the absorption spectra of the tablets included dimensionality reduction in the dataset using PCA [36]. The concept of the method is to transform the spectral data into a new coordinate system so that the greatest variance of data at any projection falls on each coordinate in the order of filling (called the first principal component—PC1, the second principal component—PC2, etc.).

## 3. Results and Discussion

### 3.1. Terahertz Optical Properties of Lactose Monohydrate Tablets

At the first stage of work on the identification of the compositions of lactose monohydrate powders, the optimal macroscopic parameters of the tablets were selected based on their optical properties in the THz frequency range. In the manufacture of tablets, special attention was paid to the preservation of the structure of the powders, therefore, a pressure of 245 MPa was chosen, at which it was possible to create and maintain a stable shape but without crushing the structure of the powders (with too weak compression, the tablet crumbled, with too strong pressure—the structure of the powder was disturbed). Having established an acceptable range of tablet density (from 1.31 to 1.39 mg·mm${}^{-3}$), a selection of tablet thickness range was carried out, which influenced the intensity of the absorption bands of lactose with different compositions. Thus, each powder composition was compressed into a tablet with a thickness of 0.91 to 3.33 mm. The obtained tablets had a stable shape, and the THz optical properties of the tablets had clear representation of the features of the lactose formulations.

**Figure 2:**
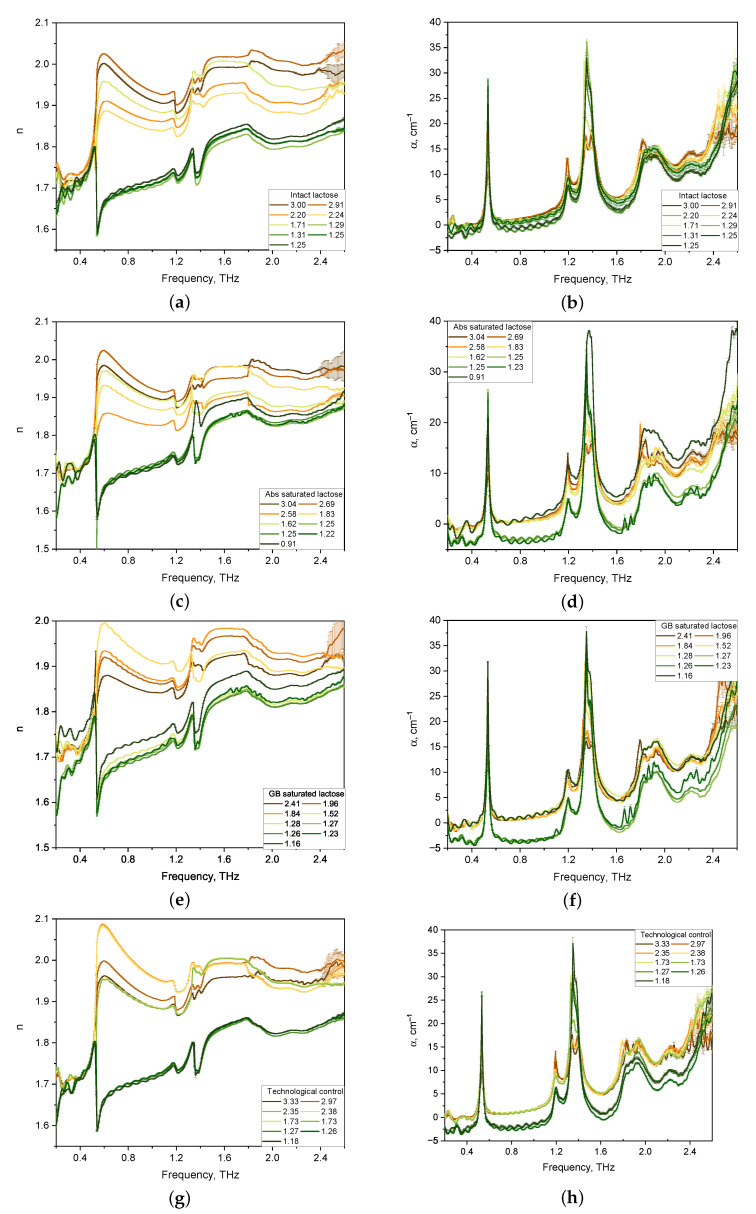
Refractive index (**a**,**c**,**e**,**g**) and absorption coefficient (**b**,**d**,**f**,**h**) spectra in the THz frequency range of lactose tablets with different formulations of various thickness (mm): intact lactose (**a**,**b**); Abs saturated lactose (**c**,**d**); GB saturated lactose (**e**,**f**); Technological control (**g**,**h**).

The spectra of optical properties of 36 lactose tablets of four formulations were investigated in the range from 0.2 to 2.6 THz. The results for the refractive index and absorption coefficient of the lactose formulations are shown in Figure 2.

Considering the absorption coefficient spectra of the tablets presented in Figure 2b,d,f,h, we note that for all tablet compositions, pronounced absorption bands of lactose were revealed, the maxima of which were located at 0.53, 1.38, and 1.82 THz. In addition, the main locations of the anomalous dispersion of lactose in the refractive index spectra of tablets of all compositions at 0.53, 1.38, and 1.82 THz were revealed (see Figure 2a,c,e,g).

The spectra of the absorption coefficient of thick tablets (1.5–3.0 mm) have no pronounced main lactose bands for all four formulations. With a decrease in the thickness of lactose tablets below 1.3 mm, the intensity of the absorption bands increases. The refractive index spectra of thick tablets have distorted lines of anomalous dispersion. This effect is associated with the influence of multiple internal reflections [37] and porosity [28].

As a result, the features in the spectra of the α-anomer of lactose monohydrate were in the agreement with those considered earlier in theoretical works [38] and experimental studies [12,13,39]. According to theoretical results obtained for the lactose monohydrate model within the density functional theory approach [12,39], the 0.53–3.02 THz range of active frequencies in the absorption spectrum of lactose monohydrate corresponds to the eigenmodes of the α-lactose molecule, while the active modes of the water molecule crystallized in the monohydrate are in the frequency range of 4.08–7.13 THz. Absorption peaks and dispersion regions at frequencies of 0.53 and 1.37 THz [12,39] presumably correspond to hindered rotational modes of the α-lactose molecule in the crystal [38]. In addition, the absorption spectra of lactose contain active frequencies of low intensities, in comparison with those for the α-anomer, at frequencies of 1.2 and 1.8–2.0 THz, which correspond to the β-anomer of lactose [12,39]. In the spectra of the refractive index of lactose, the region of anomalous dispersion at a frequency of 1.2 THz is similarly present. In the frequency range of 1.8–2.0 THz, the region of anomalous dispersion is not pronounced.

**Figure 3:**
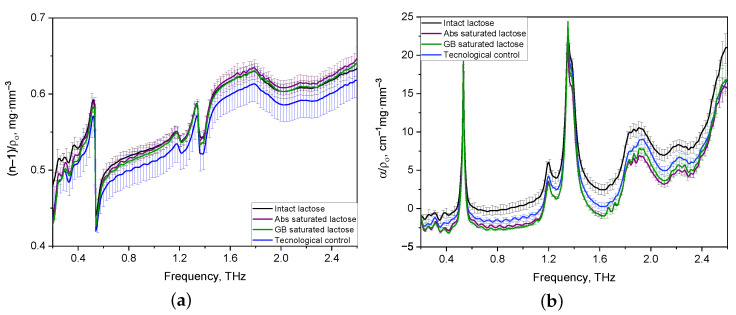
Averaged and density-normalized spectra of refractive index (**a**) and absorption coefficient (**b**) of thin lactose tablets in the THz frequency range.

From the entire set of the obtained results on analysis of THZ optical properties of formulations, a sample of spectra of tablets with thicknesses in the range of 1.2–1.3 mm was selected. The sample’s representativeness is justified by the presence of characteristics for two lactose anomers in the 0.2–2.6 THz frequency range, which are more noticeable than for THz spectra of thick tablets and tablets with a thickness of less than 1.1 mm. The THz spectra of optical properties of thin tablets (1.2–1.3 mm) were averaged for comparison and differentiation of various lactose compositions and normalized to their density [40]. The normalized spectra of the refractive indices of the formulations in the regions of normal dispersion and anomalous dispersion coincide with each other (see Figure 3a). Figure 3b shows that the normalized spectra of all lactose formulations have characteristic absorption bands of lactose, the maxima of which are located at frequencies of 0.53, 1.38, and 1.82 THz. The shape of the normalized absorption spectra of the tablets is the same for all formulations, while their difference in band intensities is noticeable, especially in the range from 1.6 to 2.0 THz. Also, differences in the intensity of spectra in the 1.8–2.0 THz region, which corresponds to the β-anomer of lactose monohydrate, are present for all tablets.

Nevertheless, the visual difference between the refractive index and absorption coefficient spectra did not provide complete information for the identification of the lactose formulations. Therefore, it was necessary to analyze the absorption spectra of lactose formulations using the Principal Component Analysis.

### 3.2. PCA of the Lactose Monohydrate Tablets

At the next stage of the study, a PCA model was built to identify the formulations of lactose based on their density-normalized absorption spectra (Figure 3b). The PCA model took into account 60 THz absorption spectra of 12 tablets with a thickness of 1.2–1.3 mm, normalized to the density of each tablet. Before the creation of the PCA model, the THz absorption spectra were preprocessed using standard normal variate transformation (SNV) and mean centering of the data [41]. The spectral range of the THz absorption spectra was scaled to 0.2 to 2.0 THz, since the high-frequency region (>2 THz) introduces a noise load.

**Figure 4:**
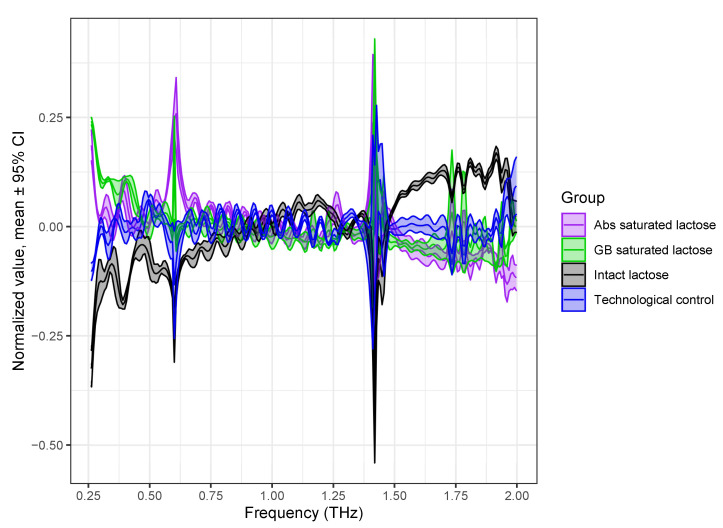
Scaled THz absorption spectra of lactose with different formulations preprocessed by normalization to SNV and mean centering of data. Spectra are presented as mean line with 95% confidence interval region.

The preprocessed spectra are shown in Figure 4. The characteristic absorption peaks of lactose with different formulations became clear, and the dependence of the spectral features on each formulation became much more apparent by preprocessing.

**Figure 5:**
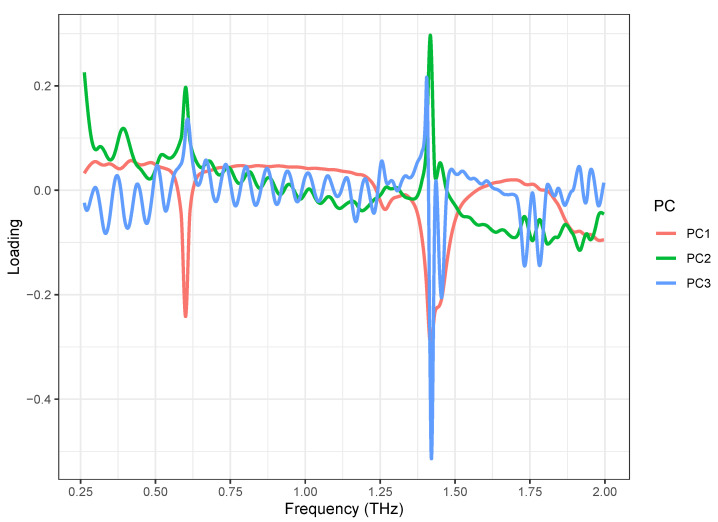
PCA loadings of first three PCs obtained from scaled spectral dataset, acquired from lactose saturated with different formulations.

The PCA loading plots are shown in Figure 5. The loading of the first principal component (PC1) (red) showed two strong negative peaks at 0.53 and 1.37 THz, which correspond to the absorption of the α-anomer of lactose monohydrate, and a weak negative peak at 1.21 THz, which corresponds to the β-anomer of lactose monohydrate. It also showed a negative band in the 1.8–2.0 THz range, which corresponds to the β-anomer of lactose monohydrate. It also showed a negative band in the 1.8–2.0 THz range, which corresponds to the β-anomer of lactose monohydrate. The loading of the second principal component (PC2) (green) showed the strongest positive peak at 1.37 THz and weak positive peaks at 0.53 THz, which correspond to the absorption of lactose monohydrate. The loading of the third principal component (PC3) (blue) showed only a strong negative peak at 1.37 THz, which corresponds to the strongest absorption of the α-anomer of lactose monohydrate. Over the entire spectral range, the loading of PC3 had a noise load. These results indicate that the PCA was successful, and the lactose formulations can be distinguished based on the first two principal components. Though PC1 and PC2 loadings look mostly mirrored, the significant differences can be observed at a band around 1.75 THz, which means the samples separated based on PC2 mostly differ in that range.

**Figure 6:**
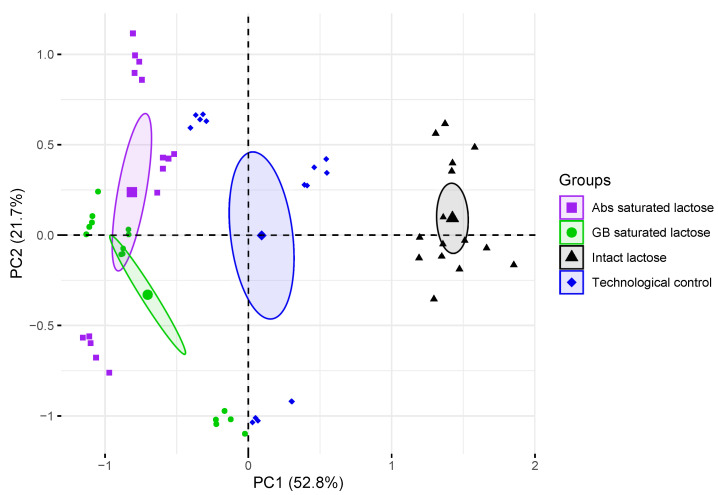
Projection of density-normalized absorption spectra of control tablets and lactose monohydrate saturated with dilutions of substances onto subspace of the first and second principal components (PC1, PC2). Enlarged points denote centroid (average coordinate) of corresponding group, ellipses outline the 95% confidence interval for centroid estimation.

The PCA model of formulations of lactose monohydrate shows that the first three main components, i.e. PC1, PC2, and PC3, contain most of the variance of spectral data with distributions of 52.8, 21.7, and 10.6% respectively. The results of the identification model are shown in Figure 6. Groups of THz absorption spectra of lactose tablets with different formulations were separated on the basis of the PCA model based on two principal components. Groups of THz absorption spectra of intact lactose, technological control and saturated lactose differed mainly in PC1, while the differences between the groups of Abs-saturated lactose and GB-saturated lactose mainly affected PC2. Based on the distribution of lactose compositions, PC1 (horizontal axis) allows to discriminate presence of a substance in solution, used for saturation. Also, groups of THz absorption spectra of intact lactose and technological control were separated in PC1 region. PC2 (vertical axis) allows to discriminate type of a substance, which high-diluted solution was used for lactose saturation. Since the THz absorption spectra of the tested lactose samples is possible to separate in the space of the main components, therefore the properties of lactose in these samples are different.

## 4. Conclusions

In this work, THz spectroscopy was combined with PCA to compare and identify four compositions of lactose monohydrate powders: intact sample and crystalline lactose saturated with a diluted solution of glycine buffer, a diluted solution of antibodies to IFN-γ, or diluted water. To study the THz optical properties of lactose monohydrate formulations, tablets were produced that had a stable shape. The THz spectra of the optical properties of tablets with a thickness from 1.50 to 3.00 mm demonstrated interference distortion of the main lactose bands due to the presence of multiple reflections. Therefore, to identify the composition of lactose, tablets with a thickness of 1.2–1.3 mm were selected, which had pronounced features of lactose in their THz optical properties. To compare the THz spectra of the optical properties of lactose compositions, the spectra were normalized to the tablet density and averaged. The results obtained revealed the main indicators of the presence of α- and β-anomers of lactose monohydrate (absorption peaks at 0.53, 1.38 THz for α-lactose and 1.2, 1.8–2.0 THz for β-lactose). Similar features were revealed in the refractive index spectra in the range of 0.4–1.6 THz. Differences in the compositions of lactose monohydrate were identified in the 1.82–2.00 THz range of absorption spectra (for β-lactose).

Using the PCA method, we showed differences in the THz absorption spectra based on two principal components. These differences have not been identified by visual analysis. Analysis of the THz absorption spectra of samples involved reducing the size of the feature space using PCA. Groups of THz absorption spectra of Abs saturated lactose and GB saturated lactose noticeably differ both from the group of intact lactose and the group of technological control spectra in the PC1 region. Groups of THz absorption spectra of intact lactose and technological control can also be separated in the PC1 region. Separation of lactose groups saturated with solutions of multiple dilutions of a substance (antibodies or glycine buffer) occurred in the PC2 region. The analysis revealed that, the properties of lactose saturated with solutions of multiple dilutions of a substance (antibodies or glycine buffer) differ both from the properties of lactose subjected to a similar technological procedure, but without adding a substance (technological control), and from the properties of intact lactose, not subjected to any treatment. In addition, the properties of lactose saturated with solutions of high dilutions of various substances (antibodies or glycine buffer) differ from each other.

The methods applied in this study are of practical use in pharmaceuticals and can be beneficial for the quality control of the medications based on the technology of repeated dilution of an original substance, since they allow to distinguish lactose saturated with dilutions of a substance (antibodies or glycine buffer) from controls that do not contain a substance. The next step in multivariate data analysis should be aimed at the collection of sufficient data on powder formulations of lactose monohydrate.

## Data Availability

Not applicable.

## Sample Availability

Samples of lactose monohydrate powders are available from OOO “NPF “MATERIA MEDICA HOLDING” (Moscow, Russia).

## Abbreviations

The following abbreviations are used in this manuscript:

UV	Ultraviolet
THz	Terahertz
THz-TDS	Terahertz time-domain spectroscopy
SNR	Signal-Noise Range
PC	Principle component
PCA	Principle Component Analysis
Abs	Antibodies
INF-γ	Interferon-gamma
GB	Glycin buffer
